# Assessment of the Technical Interpretability of Prehospital ECGs Performed by Medical and Nonmedical Teams in a French Emergency Medical System: A Descriptive Pilot Study

**DOI:** 10.1155/emmi/7380174

**Published:** 2026-05-20

**Authors:** Damien Duval, Delphine Douillet, Dominique Savary

**Affiliations:** ^1^ Department of Emergency Medicine, Angers University Hospital, Angers, France; ^2^ UMR MitoVasc, CNRS 6015–INSERM 1083, University of Angers, Angers, France, univ-angers.fr; ^3^ Inserm EHESP–UMR_S1085, Institut de Recherche en Santé, Environnement et Travail (IRSET), Angers, 49000, France

**Keywords:** ambulance technicians, electrocardiography, emergency medical services, mobile intensive care unit, prehospital care

## Abstract

**Background:**

In prehospital emergency care systems, early electrocardiogram (ECG) acquisition is essential for patient triage and management. In France, nonmedical personnel, such as private ambulance technicians and firefighters, were authorized to perform ECGs. While ECGs are crucial for diagnosis, the technical quality and interpretability of these ECGs when transmitted to emergency medical dispatch centers remain uncertain, especially with newly trained nonmedical personnel for emergency medical services (EMS) (private ambulance drivers and public firefighters). This study aimed to assess the technical interpretability of prehospital ECGs transmitted to the emergency dispatch center, according to the type of prehospital team performing ECG acquisition.

**Methods:**

A retrospective observational study was conducted at Angers Emergency Medical Dispatch Centers, France. Adult patients who underwent prehospital ECG acquisition by either mobile intensive care units (MICUs) or nonmedical EMS teams were included. The primary endpoint was the technical interpretability of ECGs based on predefined core technical criteria derived from guideline‐based ECG acquisition standards, including patient identification, recording speed and calibration, baseline stability, and absence of interference by the MICUs or nonmedical team. Precordial R‐wave transition was analyzed separately as an exploratory marker of potential electrode placement issues. To assess this outcome, all ECGs were analyzed by two independent emergency physicians blinded to clinical data and team type, with consensus adjudication in case of disagreement.

**Results:**

A total of 149 prehospital ECGs were analyzed, including 125 performed by MICU teams and 24 by nonmedical EMS. In a sensitivity analysis excluding ECGs with ST‐elevation myocardial infarction or conduction abnormalities and removing the R‐wave transition criterion from the endpoint, overall interpretability increased to 73.7% (95% CI: 65.1%–80.8%, *n* = 87/118). No statistically significant difference was observed between MICU teams (73.7%; 95% CI: 64.3%–81.4%, *n* = 73/99) and nonmedical EMS (73.7%; 95% CI: 51.2%–88.2%, *n* = 14/19) (RR 1.00; 95% CI: 0.75–1.34; *p* = 1). Normal R‐wave progression was frequently observed (53%) and was analyzed as an exploratory finding.

**Conclusion:**

In this prehospital setting, less than half of ECGs met predefined composite technical interpretability criteria. However, these criteria reflect a strict technical assessment and should not be directly equated with clinical usefulness. No significant difference was observed between medical and nonmedical teams, although the study was underpowered and estimates were imprecise. These findings highlight the technical challenges of ECG acquisition in prehospital emergency care and support the need for prospective studies to optimize training, standardization, and quality assurance. Larger prospective studies with balanced group sizes are needed to evaluate the clinical impact of prehospital ECG acquisition by nonmedical personnel and to formally assess comparative performance.

## 1. Introduction

Chest pain is a common symptom that affects up to 40% of individuals over a lifetime [1]. Prompt assessment of cardiovascular causes, especially acute coronary syndrome (ACS) and pulmonary embolism, is essential because of their high mortality and time‐sensitive management [2]. Chest pain is the second most common reason for emergency calls [3]. A total of 84% of patients calling for chest pain have a nonmedical emergency medical service (EMS) personnel, and 45% of these patients are attended by a mobile intensive care unit (MICU) with emergency physicians [4]. However, 60%–90% of these cases are benign, and most cases of chest pain are noncardiovascular [5]. Any suspicious chest pain should ideally be assessed with a clinical examination and an electrocardiogram (ECG). In the prehospital setting, performing an ECG is a key step in the management of patients presenting with cardiovascular symptoms [6].

**FIGURE 1:**
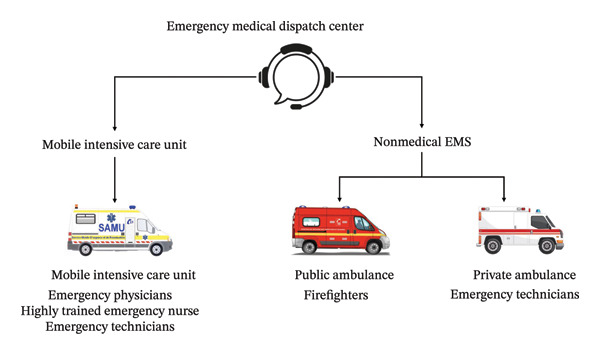
Schematic representation of the French prehospital emergency medical system. The emergency dispatch center can activate a nonmedical EMS for basic life support (private ambulance technicians or public firefighters) and/or a mobile intensive care unit (MICU) for advanced life support. Each MICU is composed of a minimum of an ambulance technician and a trained nurse, who perform ECGs, and may be accompanied by an emergency physician. EMS: emergency medical services.

In France, the coordination of out‐of‐hospital emergencies is managed by the Service d’Aide Médicale d’Urgence (SAMU). Each SAMU includes a medical dispatch center, dispatching physicians, and MICUs. The dispatching physician determines the most appropriate response according to two levels of emergency (Figure 1). The first level involves private ambulance personnel and firefighters, who provide basic life support (BLS). The second level corresponds to the deployment of a medicalized unit for advanced life support (ALS). Each MICU is staffed by a senior emergency physician, a nurse, and a trained ambulance driver. These units are capable of performing all advanced resuscitation procedures, including clinical examination, ECG, endotracheal intubation, and cardiopulmonary resuscitation [7]. The performance of ECG in the MICU team can be carried out by a registered and trained nurse on medical prescription (Article R4311‐7 of the French Public Health Code). To optimize MICU dispatch and address shortages in certain regions of France, a ministerial decree (No. 2022‐631 of April 22, 2022) extended the authorization to perform ECG to nonmedical EMS personnel, such as private ambulance technicians and public firefighters, in prehospital emergency medical care. Each private ambulance technician received a comprehensive training provided by the Emergency Care Training Center of Maine‐et‐Loire (CESU49) for the performance of ECGs. Angers EMS covers both an urban center (Angers city) and semirural surrounding areas, with a mixed public–private prehospital response system.

The performance and interpretability of ECGs are based on several criteria, such as the number of leads, the amplitude and recording speed, the absence of interference, the baseline stability, and particularly the correct placement of electrodes [8, 9]. The performance of an ECG, particularly electrode placement, is based on a standardized layout that ensures reproducible analysis [10]. Electrode positioning is directly linked to the reliability and integrity of the signals, which are interpreted to guide diagnosis and treatment [11, 12]. ECG signals recorded with incorrectly placed electrodes can lead to significant errors and false diagnoses [13]. A study by Bond et al. in 2012 showed that improper electrode placement could result in misdiagnosis in 17%–24% of cases [14].

Given the necessity of relying on accurate data to determine patient management, it is essential that the ECG be performed in a way that ensures that it is fully interpretable by an emergency physician. This study aimed to assess the interpretability of prehospital ECGs transmitted to emergency medical dispatch centers and performed by both medical and nonmedical teams.

## 2. Methods

### 2.1. Prehospital French Medical System and Patients

The study was designed as a descriptive pilot study conducted during the early phase of implementation of ECG acquisition by nonmedical EMS personnel. This study was conducted from April 1 to April 30, 2024, on a cohort of adult patients who underwent an ECG during prehospital care, either by a MICU (medicalized or not) or by a nonmedical team (ambulance technicians or firefighters). The one‐month period corresponds to the initial phase of deployment of ECG‐trained nonmedical EMS, explaining the limited timeframe.

The SAMU corresponds to a base station equipped with one or more MICUs, each composed of an ambulance driver, a nurse, and a senior emergency physician, together forming the complete medical care team. Some interventions do not require the presence of an emergency physician and are carried out solely by ambulance technicians or a nurse (paramedical MICU), under the supervision of the dispatching physician, who monitors the situation remotely through transmitted vital signs (Figure 1, Supporting Appendix 1). These teams are limited in the medical procedures they are authorized to perform on the patient. Prehospital interventions by a medicalized MICU conducted outside a healthcare facility are considered primary interventions. Interhospital transfers are classified as secondary interventions, while intrahospital transfers within the same facility are tertiary interventions. It should be noted that secondary and tertiary transfers may be either medical or paramedical, depending on the patient’s condition and resource availability. For clarity, the term “nonmedical EMS” refers to private ambulance technicians and public firefighters, whereas “MICU teams” refers to MICUs, which may be medicalized or paramedical depending on physician presence.

ECGs performed by MICU teams were carried out at the request of the attending physician or, in some cases, upon prescription by the dispatching physician when involving a MICU crew without an emergency physician or nonmedical EMS. Each ECG was linked to a unique regulation file, ensuring patient identification. The study database was extracted from medical dispatch files, from which the following data were extracted: sex, age, type of intervention, reason for the emergency call, final diagnosis, patient’s destination upon conclusion of care, and the corresponding ECG. Patients were included in the study if they had contacted the emergency dispatch center and received an ECG as part of their care by an MICU or nonmedical EMS. Patients who did not undergo an ECG were excluded. ECGs were transmitted to the emergency dispatch center via email and subsequently incorporated into the emergency medical dispatch center’s records. Definitions of variables and data processing steps are detailed in Supporting Figure 1.

### 2.2. Training of Nonmedical EMS Personnel and ECG Acquisition Condition

Nonmedical EMS personnel (ambulance technicians) involved in ECG acquisition underwent a standardized training program delivered by the Emergency Care Training Center (CESU49) prior to field implementation. This training consisted of a one‐day session combining theoretical instruction and hands‐on practice. The theoretical component included the details of the training material and basic principles of electrocardiography, standard ECG acquisition procedures, and recognition of common technical errors (Supporting Figure 2). The practical component focused on correct electrode placement using anatomical landmarks, proper patient positioning, and acquisition under simulated prehospital conditions. Training sessions included supervised practice with immediate feedback from instructors. At the time of the study, no formal competency reassessment or certification process was implemented following the initial training. Refresher training sessions are planned every four years as part of the national EMS training framework. As for the firefighters, the training was provided by the Medical and Rescue Health Service under the same conditions as for the private ambulance technicians.

Prehospital ECGs were acquired using standard portable 12‐lead ECG devices available within the EMS system. MICUs were equipped with Corpuls 3T monitors (Corpuls, Germany), while nonmedical EMS personnel used multifunction Schiller devices (Schiller AG, Switzerland). ECG acquisition was performed under real‐life prehospital conditions, including private homes, public environments, and during patient transport. As a result, recordings were subject to variable environmental conditions such as patient positioning constraints, movement, and potential electrical interference. Due to the retrospective design of the study, detailed technical specifications (including software version, filter settings, and sampling rate) were not consistently available for all recordings and were therefore not systematically analyzed.

### 2.3. Primary Outcome

The primary aim was to evaluate the interpretability criteria of ECGs performed in prehospital settings and transmitted to emergency medical dispatch centers either by MICU or nonmedical EMS (private ambulance technicians and firefighters). The primary outcome was the proportion of ECGs meeting all predefined technical interpretability criteria. The technical interpretability criteria used in this study were defined a priori with reference to established electrocardiographic standards, primarily the AHA/ACC/HRS recommendations and the AHA/ACC scientific statements on ECG standardization and acquisition quality. These documents were used as the main methodological framework for identifying core technical elements required for a readable and standardized 12‐lead ECG recording [11]. The criteria used for ECG interpretation in the study included the following: (1) patient identification (ensuring the correct recording of the patient’s first and last name), (2) ECG parameters (verification of a recording speed of 25 mm/s and a calibration of 10 mm/1 mV), (3) baseline stability (assessment of the stability of the isoelectric line), and (4) absence of interference (ensuring that the ECG is free from artifacts or parasitic signals). Briefly, patient identification was retained as a prerequisite for safe interpretation and traceability of the recording; standard paper speed and voltage calibration were required to ensure waveform comparability; baseline stability was used to reflect recording quality; and absence of artefact was used to indicate sufficient signal quality for visual interpretation. This concordance rate is considered reliable if all predefined interpretability criteria are met for analysis. All ECGs were independently reviewed by two emergency physicians using predefined interpretability criteria. Reviewers assessed the ECGs separately before consensus adjudication of disagreements. Full blinding to the performing team was not possible because some ECG metadata or formatting features could indirectly suggest the origin of the recording. This incomplete blinding may have influenced the subjective assessment of certain criteria and was therefore considered a potential source of observer bias. In case of disagreement, both reviewers jointly reevaluated the discordant criterion and reached a consensus decision. No third reviewer was involved. Both work at emergency medical dispatch centers, the MICU, and the emergency department. The primary endpoint was restricted to technical ECG interpretability according to predefined acquisition‐quality criteria. The study was not designed to assess diagnostic accuracy, clinical decision‐making, patient triage performance, reperfusion delays, or patient outcomes.

### 2.4. Secondary Outcome

The secondary outcomes were interpretability rates among MICU and nonmedical EMS. Each criterion of ECG interpretability is compared between the two groups. Precordial R‐wave progression was used as a surrogate marker of correct electrode placement, as recommended in previous studies and standard ECG interpretation frameworks [11]. The proportion of ECGs meeting predefined technical criteria was described for each group with corresponding 95% confidence intervals. Analyses of individual interpretability criteria were considered exploratory, without adjustment for multiple comparisons.

### 2.5. Statistical Analysis

Baseline characteristics are presented as counts (percentages), means ± standard deviations, or medians with interquartile ranges, as appropriate. Missing data not available from medical records were excluded from analysis. This study was conducted as a pilot analysis during the early implementation phase of prehospital ECG acquisition by nonmedical EMS personnel; therefore, no formal sample size calculation was performed, and all eligible ECGs during the study period were included.

The primary outcome was analyzed as a binary variable. Between‐group comparisons were performed using chi‐square or Fisher’s exact tests, as appropriate. A two‐sided *p* value < 0.05 was considered statistically significant, and results are reported with 95% confidence intervals. Given the pilot design and marked group imbalance, wide confidence intervals were expected. Accordingly, estimates were interpreted as descriptive measures of effect size and uncertainty rather than as evidence for definitive between‐group differences. This level of precision was considered acceptable for exploratory purposes but insufficient for formal equivalence or noninferiority assessment. Because no universally accepted threshold exists for the composite endpoint, an a priori descriptive benchmark was defined. For key technical criteria related to ECG readability (speed/calibration, baseline stability, and absence of major artefact), a compliance rate ≥ 85% was considered pragmatically acceptable based on expected standards in routine clinical practice. This threshold was used for descriptive interpretation only and not for hypothesis testing. Interobserver agreement was assessed using Cohen’s kappa coefficient at the global level. Criterion‐specific kappa estimates were not reported because sparse discordant observations and ceiling effects would likely produce unstable estimates in this small sample. This was a deliberate methodological choice rather than an omission. Statistical analyses were performed using R software (R Core Team 2014 R: A Language and Environment for Statistical Computing. R Foundation for Statistical Computing, Vienna, Austria).

### 2.6. Ethical Consideration

This study was conducted in accordance with the ethical standards of the institutional review board and the principles of the Declaration of Helsinki. All relevant parts of the study, including patients’ clinical data and access to these data, were reviewed and approved by the Ethics Committee of Angers University Hospital (No. 2024‐086). There was no need for patients’ informed consent because the study was based on ECGs and medical records already examined by the physician prehospitally. The collected information was used only for clinical research purposes, with access limited to study group staff. Patients’ identities were not shared, and cases were coded numerically. Reporting follows STROBE and RECORD recommendations (Supporting Figures 3 and 4).

## 3. Results

### 3.1. Study Population

**FIGURE 2:**
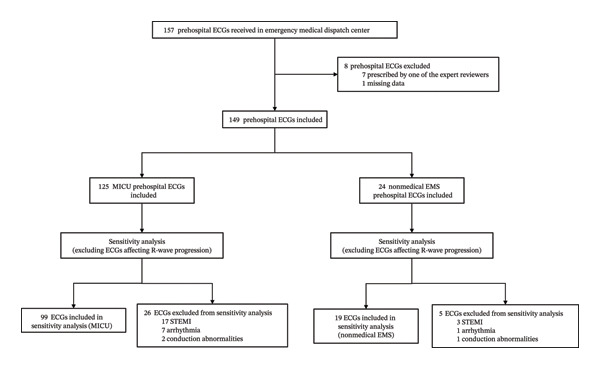
Flow diagram of prehospital ECG selection and analysis. A total of 157 prehospital ECGs were received, of which 149 were included in the primary analysis after exclusion of 8 ECGs (7 prescribed by an expert reviewer and 1 with missing data). ECGs were stratified by performing team: mobile intensive care unit (MICU) (*n* = 125) and nonmedical emergency medical services (EMS) (*n* = 24). A sensitivity analysis was conducted by excluding ECGs with conditions likely to affect R‐wave progression (STEMI, arrhythmia, or conduction abnormalities) and removing the R‐wave transition criterion. This resulted in 99 ECGs in the MICU group and 19 in the nonmedical EMS group. This diagram distinguishes the primary analysis from the sensitivity analysis, in which R‐wave progression was considered an exploratory marker rather than a core technical criterion.

In April 2024, 157 patients had an ECG outside of the hospital, and only 149 patients were included in this study. Seven patients were excluded because of an ECG prescription by one of these experts and 1 because of a lack of data. A total of 149 patients were included, with 125 (84%) in the MICU and 24 (16%) in the nonmedical EMS (Figure 2). All ECGs acquired by nonmedical EMS during the study period were obtained following a dispatch physician’s request, reflecting targeted use rather than systematic ECG acquisition. The patients’ characteristics are summarized in Table 1. The median age was 64 years ± 17. Patients were predominantly male (*n* = 88, 59%). Most of the ECGs came from a MICU medical team according to primary MICU prehospital response (*n* = 93, 62.4%), followed by secondary MICU prehospital response (*n* = 10, 6.7%) and tertiary MICU prehospital response (*n* = 5, 3.5%). With respect to the first‐level response, 20 (13.4%) took place during the study period by private ambulance technicians and 3 (2%) by public firefighters. Among all the level responses, the main reason for calling the emergency medical dispatch centers was chest pain suggestive of ACS (*n* = 62, 41.6%), followed by atypical chest pain (*n* = 15, 10.1%), dyspnea (*n* = 13, 8.7%), arrhythmias (*n* = 13, 8.7%), and syncope (*n* = 8, 8.4%). The main destination for patients was the emergency department (*n* = 56, 37.6%), followed by the cardiac intensive care unit (*n* = 53, 35.6%) and then coronary angiography (*n* = 18, 12.1%). Notably, a total of 5 ECGs were performed in the prehospital setting by nonmedical EMS, which led to the deployment of a MICU medical team for the urgent management of myocardial infarction (Table 1).

**TABLE 1:** Patients’ characteristics.

Patients’ characteristics	Total *n* = 149 (%)	MICU teams *n* = 125 (%)	Nonmedical EMS *n* = 24 (%)
Age, mean (SD), years	64 (17)	64 (17)	58 (21.5)
Sex, male (%)	88 (59)	76 (61)	12 (50)
Type of intervention, *n* (%)			
Primary	93 (62.4)	93 (74.4)	—
Secondary	10 (6.7)	10 (8.0)	—
Tertiary	5 (3.4)	5 (4.0)	—
Paramedic secondary	14 (9.4)	14 (11.2)	—
Paramedic tertiary	3 (2)	3 (2.4)	—
Ambulance technicians	20 (13.4)	—	20 (83.3)
Firefighter rescuers	3 (2.7)	—	4 (16.7)
Type of emergency calling, *n* (%)			
Chest pain suggestive of ACS	62 (41.6)	58 (46.4)	6 (25)
Dyspnea	13 (8.7)	10 (8.0)	3 (12.5)
Arrhythmia	13 (8.7)	12 (9.6)	1 (4.2)
Acute heart failure	13 (8.7)	13 (10.4)	—
Syncope	8 (5.4)	7 (5.6)	1 (4.2)
Neurology	7 (4.7)	7 (5.6)	—
Others	8 (5.4)	7 (5.6)	1 (4.2)
Traumatology	6 (4)	5 (4.0)	1 (4.2)
Atypical chest pain	15 (10.1)	4 (3.2)	11 (45.8)
Cardiac arrest	3 (2)	3 (2.4)	—
Main destination, *n* (%)			
Emergency department	56 (37.6)	41 (32.8)	15 (62.5)
Cardiology intensive care unit	53 (35.6)	48 (38.4)	5 (20.8)
Coronary angiography	18 (12.1)	16 (12.8)	2 (8.3)
Intensive care unit	11 (7.4)	11 (8.8)	—
Trauma center	4 (2.7)	4 (3.2)	—
Leave on site	4 (2.7)	2 (1.6)	2 (8.3)
Radiology	1 (0.7)	1 (0.8)	—
Medicine	3 (2)	3 (2.4)	—
Diagnostic, *n* (%)			
STEMI	20 (13.4)	17 (13.6)	3 (12.5)
NSTEMI	13 (8.7)	13 (10.4)	0 (0.0)
Atypical chest pain	25 (16.7)	16 (12.8)	9 (37.5)
Cardiac conduction disease	8 (5.3)	7 (5.6)	1 (4.2)
Arrhythmia	3 (2.0)	2 (1.6)	1 (4.2)
Cardiac arrest	2 (1.3)	2 (1.6)	0 (0.0)
Acute heart failure	10 (6.7)	10 (8.0)	0 (0.0)
Aortic dissection	2 (1.3)	2 (1.6)	0 (0.0)
Syncope	2 (1.3)	1 (0.8)	1 (4.2)
Myocarditis	2 (1.3)	2 (1.6)	0 (0.0)
Dyspnea	7 (4.7)	5 (4.0)	2 (8.3)
Traumatology	6 (4.0)	5 (4.0)	1 (4.2)
Infectious disease	6 (4.0)	4 (3.2)	2 (8.3)
Nephrology	4 (2.7)	3 (2.4)	1 (4.2)
Neurology	13 (8.7)	13 (10.4)	0 (0.0)
Hepatogastroenterology	7 (4.7)	7 (5.6)	0 (0.0)
Others	17 (11.4)	14 (11.2)	3 (12.5)
Psychiatry	2 (1.3)	2 (1.6)	0 (0.0)

*Note:* MICU teams correspond to ambulance technicians and emergency nurses, with or without an emergency physician. Paramedical MICU refers exclusively to inter‐ or intrahospital transfers without an emergency physician. Nonmedical EMS corresponds to private ambulance technicians or public firefighters. ECG: electrocardiogram.

Abbreviations: ACS, acute coronary syndrome; MICU, mobile intensive care unit; NSTEMI, non–ST‐elevation myocardial infarction; SD, standard deviation; STEMI, ST‐elevation myocardial infarction.

### 3.2. Primary Outcome

**FIGURE 3:**
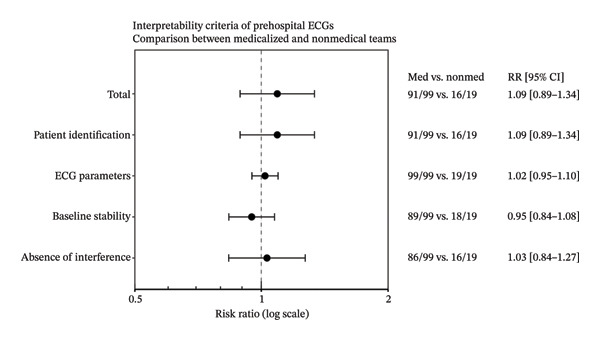
Forest plot of compliance with predefined technical ECG criteria in the prehospital setting. Forest plot showing the comparison of ECG interpretability criteria between medicalized and nonmedical prehospital teams. Results are expressed as risk ratios (RRs) with 95% confidence intervals (CIs). The vertical dashed line represents the null value (RR = 1), indicating no difference between groups. Each point estimate corresponds to the RR for a given interpretability criterion, while horizontal lines indicate the associated 95% CI. Values to the right of the line (RR > 1) favor medicalized teams, whereas values to the left (RR < 1) favor nonmedical teams. The right panel displays the number of interpretable ECGs over the total number of ECGs in each group (med vs. nonmed), along with the corresponding RR and 95% CI. Overall, no statistically significant differences were observed between groups for any interpretability criterion, as all confidence intervals include the null value.

**FIGURE 4:**
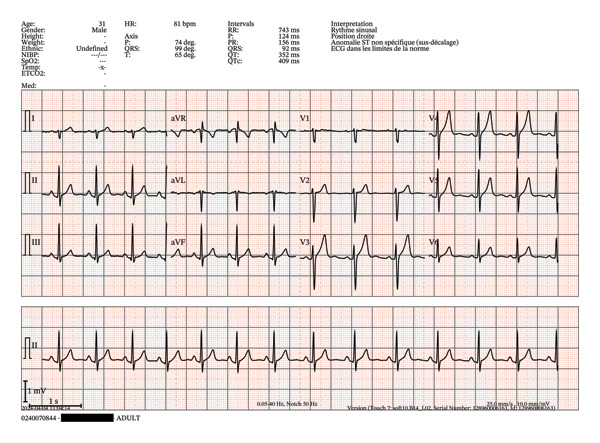
Example of a normal ECG by ambulance technicians. Normal prehospital 12‐lead ECG fulfilling all interpretability criteria, including correct patient identification, standard recording speed and calibration (25 mm/s, 10 mm/mV) with visible scale bars, 0.05–40 Hz, notch 50 Hz, stable isoelectric baseline, absence of interference, correct lead polarity, and harmonious R‐wave progression from V2 to V4, consistent with appropriate precordial electrode placement. Blackbox masks the real name of the patient.

**FIGURE 6:**
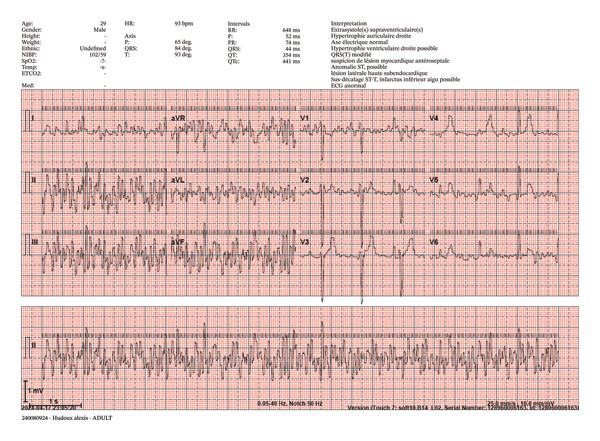
Example of an abnormal ECG by ambulance technicians. Representative 12‐lead ECG acquired in the prehospital setting, demonstrating multiple technical and interpretative abnormalities. The recording was obtained under standard settings (25 mm/s, 10 mm/mV) with visible scale bars and shows significant baseline instability and motion‐related artefacts affecting several leads, particularly in the limb leads. Precordial leads display heterogeneous R‐wave progression, which may reflect suboptimal electrode placement and/or underlying clinical conditions. ST‐segment abnormalities are also observed but should be interpreted with caution, given the presence of artefacts and signal noise. This example illustrates the challenges of ECG acquisition in real‐life prehospital conditions, where environmental factors (patient movement, positioning, and transport conditions) may compromise signal quality and limit interpretability. All patient‐identifying information has been removed or masked.

In a sensitivity analysis performed after excluding ECGs with STEMI or conduction abnormalities and removing the R‐wave transition criterion, the rate of compliance with interpretability criteria was 73.7% (95% CI: 65.1%–80.8%, *n* = 87/118) for all ECGs (Table 2). Although the overall interpretability rate increased compared with the primary analysis, no statistically significant difference was observed between MICU teams (73.7%; 95% CI: 64.3%–81.4%, *n* = 73/99) and nonmedical EMS (73.7%; 95% CI: 51.2%–88.2%, *n* = 14/19) (Table 2). For other interpretative elements, compliance rates ranged from 90.7% (95% CI: 83.9%–95.3%, *n* = 107/118) for patient identification to 86.4% (95% CI: 78.9%–92.0%, *n* = 102/118) for absence of interference. The predefined technical criteria, derived from guideline‐based ECG acquisition standards, were applied consistently across both groups. Proportions varied across criteria, with high compliance observed for some technical aspects such as patient identification and absence of interference, while other criteria, particularly those related to precordial lead assessment, showed lower compliance (Table 2). Compliance with each predefined technical criterion is presented in Figure 3 as a forest plot with 95% confidence intervals for both MICU and nonmedical EMS groups. High compliance rates were observed for patient identification and the absence of interference. Exploratory analyses showed lower compliance for criteria related to precordial lead assessment. No statistically significant differences were observed between groups across individual criteria, although confidence intervals were wide, reflecting limited precision. A representative example of prehospital ECGs illustrating normal (Figure 4) and common acquisition errors, including abnormal R‐wave transition related to electrode misplacement (Figures 5 and 6).

**TABLE 2:** Primary and secondary outcomes after exclusion of ECGs with STEMI or cardiac conduction abnormalities and removal of the R‐wave transition criterion.

	**Total *n* = 118** **(%) [95% CI]**	**MICU teams *n* = 99** **(%) [95% CI]**	**Nonmedical EMS *n* = 19** **(%) [95% CI]**	**RR** **(%) (95% CI)**	***p* value**

Primary outcome					
ECG interpretation	87 (73.7) 65.1%–80.8%	73 (73.7) 64.3%–81.4%	14 (73.7) 51.2%–88.2%	1.0 (0.75–1.34)	1
Secondary outcomes					
Patient identification	107 (90.7) 83.9%–95.3%	91 (91.9) 84.7%–96.4%	16 (84.2) 60.4%–96.6%	1.09 (0.89–1.34)	0.381
ECG parameters	118 (100) 96.9%–100.0%	99 (100) 97.1%–100.0%	19 (100) 82.4%–100.0%	1.02 (0.95–1.10)	1
Baseline stability	107 (90.7) 83.9%–95.3%	89 (89.9) 82.2%–95.0%	18 (94.7) 74.0%–99.9%	0.95 (0.84–1.08)	1
Absence of interference	102 (86.4) 78.9%–92.0%	86 (86.9) 78.6%–92.8%	16 (84.2) 60.4%–96.6%	1.03 (0.84–1.27)	0.721

*Note:* MICU teams correspond to ambulance technicians and emergency nurses, with or without an emergency physician. Nonmedical EMS corresponds to private ambulance technicians or public firefighters. A chi‐square or Fisher’s exact test was used for the primary outcome. ECG: electrocardiogram.

Abbreviations: EMS, emergency medical services; MICU, mobile intensive care unit.

FIGURE 5(a) Example of prehospital ECG—frontal leads. Representative prehospital 12‐lead ECG focusing on limb (frontal) leads (I, II, III, aVR, aVL, and aVF), recorded under standard conditions (25 mm/s, 10 mm/mV) with visible scale bars. All patient‐identifying information has been removed or masked. (b) Representative prehospital ECG showing precordial leads (V1–V6), recorded under standard settings (25 mm/s, 10 mm/mV). All patient‐identifying information has been removed.

**Figure:**
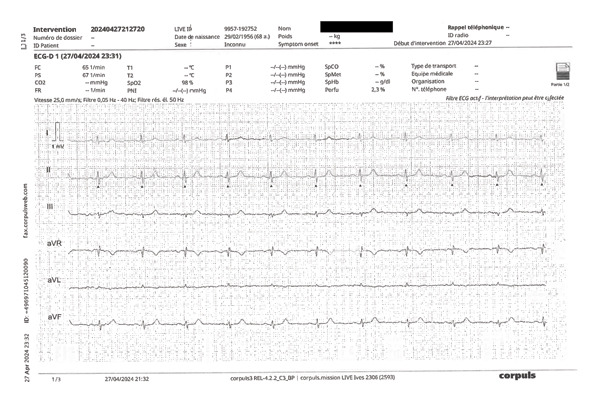
(a)

**Figure:**
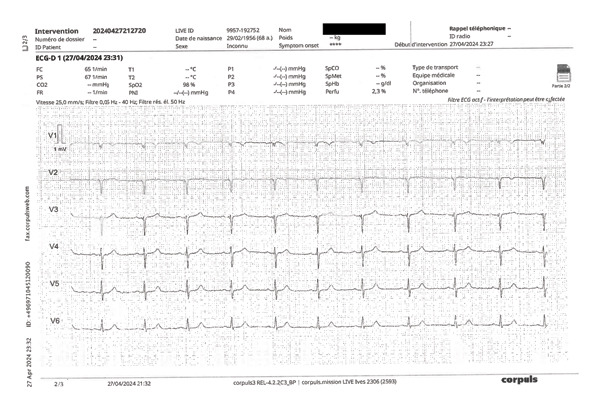
(b)

### 3.3. Secondary Outcomes

For R‐wave criteria outcome, the interpretability rates did not vary significantly between medical and nonmedical teams: 48.8% for the MICU teams and 37.5% for the nonmedical EMS (RR = 1.30; *p* = 0.428; 0.75–2.25) (Table 3). Although the point estimate suggested a higher interpretability rate in MICU teams (RR 1.30), the confidence interval was wide (95% CI: 0.75–2.25), reflecting limited statistical power due to the small number of ECGs performed by nonmedical EMS. Among exploratory criteria, abnormalities in R‐wave progression were frequently observed in leads V2–V4, with a rate of 53% (95% CI: 44.7%–61.2%, *n* = 79/149).

**TABLE 3:** Secondary outcomes.

	**Total *n* = 149** **(%) (95% CI)**	**MICU teams *n* = 125** **(%) (95% CI)**	**Nonmedical EMS *n* = 24** **(%) (95% CI)**	**RR** **(%) (95% CI)**	***p* value**

Secondary outcomes					
ECG interpretation	70 (47.0) 38.8%–55.3%	61 (48.8) 39.8%–57.9%	9 (37.5) 18.8%–59.4%	1.30 (0.75–2.25)	0.428^∗^
R‐wave transition	79 (53.0) 44.7%–61.2%	68 (54.4) 45.3%–63.3%	11 (45.8) 25.6%–67.2%	1.19 (0.75–1.89)	0.584^∗^

*Note:* MICU teams correspond to ambulance technicians and emergency nurses, with or without an emergency physician. Nonmedical EMS corresponds to private ambulance technicians or public firefighters. ECG: electrocardiogram.

Abbreviations: EMS, emergency medical services; MICU, mobile intensive care unit.

^∗^Corresponding to a Fisher’s exact test.

Interobserver agreement between the two emergency physicians was excellent, with a crude agreement of 98% and a Cohen’s kappa of 0.96 (95% CI: 0.91–1.00), indicating almost perfect agreement.

## 4. Discussion

The study revealed that less than half of prehospital ECGs met predefined technical interpretability criteria, reflecting the constraints of prehospital conditions rather than a direct measure of clinical performance. Any downstream clinical implications discussed in this manuscript should therefore be interpreted as hypothesis‐generating only. Although technical interpretability is a prerequisite for reliable ECG use, this study does not establish a direct relationship between technical quality, diagnostic performance, or patient outcomes. No statistically significant difference was observed between groups; however, this should be interpreted as the absence of evidence of a difference rather than evidence of equivalence. Given the limited sample size and wide confidence intervals, the study was not powered for definitive comparative conclusions. These findings highlight that electrode placement errors, rather than signal noise alone, represent the predominant technical limitation affecting ECG interpretability in the prehospital setting. Therefore, no difference could be demonstrated in this underpowered pilot cohort. The importance of properly performing an ECG lies in the early identification of potential abnormalities to ensure appropriate management and patient triage. To this end, all medical and paramedical staff are trained in ECG acquisition, with multiple daily practice sessions [15].

This difference between the two types of interventions may seem surprising, given that the same personnel are involved in acquiring the ECG. One possible explanation lies in the type and setting of the intervention. Indeed, paramedical interventions involve only inter‐ or intrahospital transfers, where the patient is typically well positioned and prepared under optimal conditions. In contrast, prehospital settings present challenges that can interfere with ECG acquisition—such as vehicle vibrations, outdoor environments, or suboptimal patient positions (e.g., on beds or couches at home)—which may affect the quality and interpretability of the ECG. One of the limitations of this study is the number of ECGs performed by nonmedical EMS. This low rate can be explained by the fact that training has only been provided by the Emergency Care Teaching Center since early January 2024, with a gradual increase in the number of trained personnel. Additionally, there was a high demand for ECG machines, which limited deployment within first responder organizations. This selection mechanism may limit the external validity of the findings and restrict generalization to all nonmedical prehospital interventions.

The main technical limitation observed was related to precordial lead assessment, particularly R‐wave transition, which was analyzed as an exploratory marker. Precise electrode placement during ECG acquisition is crucial, as it directly impacts the reliability and accuracy of the signals used to guide diagnosis and treatment decisions [12]. The high proportion of abnormal R‐wave transition observed in this study should not be interpreted solely as evidence of electrode misplacement. In the prehospital emergency setting, ECG acquisition is often performed under challenging conditions, including suboptimal patient positioning, environmental constraints, and time pressure during emergency care. In addition, abnormal R‐wave progression may also reflect underlying cardiac pathology rather than technical error. ECG signals recorded with improperly positioned electrodes can lead to significant interpretive errors, resulting in false‐positive diagnoses of anterior myocardial infarction, ventricular hypertrophy, ischemia, or Brugada syndrome [13]. A study by Bond et al. (2012) revealed that incorrect electrode placement could lead to misdiagnosis in 17%–24% of cases [14]. The 12‐lead ECG has limited sensitivity (30%–70%) and specificity (70%–95%) for detecting ACS. ECGs may appear normal in many ACS patients for various reasons—one of the most important being incorrect precordial lead placement [12]. Although widely used, normal R‐wave transition is known to be variable and may be influenced by factors such as body habitus, chronic lung disease, cardiac rotation, or ischemia. As a result, some ECGs with technically correct electrode placement may have been classified as noninterpretable based on this criterion. Because abnormal R‐wave progression may reflect underlying cardiac pathology rather than electrode misplacement, a sensitivity analysis excluding ECGs with STEMI or conduction abnormalities and removing the R‐wave transition criterion was performed. This analysis increased the overall interpretability rate but did not modify the absence of significant differences between medical and nonmedical teams [15].

Overall, while the ECG interpretability criteria used in this study are supported by existing standards, certain methodological assumptions—particularly regarding R‐wave transition and the use of a quality‐of‐care reference—should be interpreted cautiously. Therefore, the observed rate likely reflects a combination of technical challenges and patient‐related clinical factors rather than a pure quality‐of‐care deficit. Importantly, the interpretability criteria used in this study represent a composite technical assessment and should not be directly equated with clinical usefulness. In prehospital care, ECGs that do not fulfill all predefined technical criteria may still provide valuable diagnostic or triage information. Therefore, the observed rate of noninterpretability should not be interpreted as a direct indicator of poor performance by EMS personnel, but rather as a reflection of the complexity of ECG acquisition in emergency conditions and the limitations of the selected surrogate markers.

Multiple types of electrode misplacement, such as right arm/right leg reversal or attenuated signal amplitude, can result in misdiagnosis of ACS in healthy patients [11, 16, 17]. Therefore, proper training in ECG acquisition is essential to ensure accurate interpretation, especially considering that suspected ACS is one of the most common reasons for dispatching MICU teams. Patients with suspected ACS during the emergency call must be identified and transported to an appropriate care facility by MICU teams. Transport by the MICU medical team allows for the assessment, monitoring, and treatment of life‐threatening conditions such as arrhythmias or cardiac arrest on the road to the emergency department [18]. Prehospital acquisition of a 12‐lead ECG by trained personnel is associated with shorter reperfusion times and reduced mortality in STEMI patients [18–20]. Repeating an ECG is crucial, as it allows timely patient triage and management without delay. Several studies have shown that 8%–15% of initially normal ECGs reveal STEMI during follow‐up [22, 23]. The dispatch of a medical team remains essential, even when a first responder team has already identified abnormalities on an ECG. Patients with ACS are at the highest risk of death due to prehospital complications such as heart failure, ventricular arrhythmias, or cardiogenic shock. Therefore, it is vital to have specialized teams capable of anticipating and managing these complications and ensuring transport to a PCI‐capable center [6]. Although MICU resources are limited and alternative strategies are sometimes needed for managing chest pain in the prehospital setting, they cannot replace the expertise of a specialized medical team. These elements relate to established guideline‐based practice rather than being directly evaluated in our study. The primary endpoint of this study was technical ECG interpretability rather than clinical accuracy or patient‐centered outcomes. Therefore, although ECG interpretability is a prerequisite for accurate diagnosis and appropriate triage, this study does not allow conclusions to be drawn regarding STEMI detection, coronary angiography activation, or time‐to‐reperfusion. Any association between ECG interpretability and downstream clinical outcomes remains inferential and was beyond the scope of the present study.

The emergence of AI, particularly in the field of ECG interpretation (such as *Queen of Heart* or *PMcardio*), may change the landscape, especially for the early identification of ACS [24, 25]. If AI interpretation can achieve noninferiority compared with physicians, the use of nonmedical personnel to acquire ECGs becomes even more relevant.

### 4.1. Limitations

This study has several limitations related to its pilot design, sample size, and statistical precision. The marked imbalance between groups (118 ECGs in MICU teams vs. 19 in nonmedical EMS) reflects the early implementation phase of ECG acquisition by nonmedical personnel, during which training and equipment deployment were still ongoing. As a result, the number of ECGs in the nonmedical group was limited, leading to wide confidence intervals and reduced statistical precision. The study was therefore underpowered to detect anything other than large differences between groups. Accordingly, the absence of a statistically significant difference should be interpreted as the absence of evidence rather than evidence of equivalence.

Selection bias must also be considered. In our system, ECG acquisition by nonmedical EMS personnel was performed only upon the dispatch physician’s request, likely resulting in a selected population with higher clinical suspicion of cardiac pathology compared with the broader population encountered during BLS interventions. In addition, differences in professional experience, perceived responsibility, or engagement in ECG acquisition may have influenced performance across groups.

Full blinding of reviewers was not possible. Although assessors were not explicitly informed of the performing team, ECG layout characteristics or metadata may have suggested the origin of the recording. This may have introduced observer bias, particularly for criteria involving subjective assessment, such as baseline stability or artefact evaluation.

The study did not account for potential clustering at the team or responder level. Because responder‐level data were incomplete and the number of ECGs per cluster was limited, hierarchical modeling was not feasible. This may have led to an underestimation of uncertainty and should be considered a methodological limitation.

Interobserver agreement was assessed globally using Cohen’s kappa, but criterion‐specific kappa estimates were not reported due to sparse discordant observations and ceiling effects, which can produce unstable estimates in small samples. This was a deliberate methodological choice.

The criteria used to define ECG interpretability were based on existing standards, but some components—particularly R‐wave transition—may be influenced by both technical factors and underlying patient characteristics, and may vary between observers. Although a sensitivity analysis excluding ECGs with STEMI or conduction abnormalities and removing this criterion was performed, these results should be interpreted cautiously given the limited sample size. These limitations support interpreting the findings as exploratory and hypothesis‐generating rather than confirmatory.

Operational constraints inherent to the prehospital setting may also have affected ECG acquisition quality. Factors such as patient positioning, environmental conditions, time pressure, and competing priorities during emergency care may have contributed to variability in technical performance [26].

Finally, as this study was conducted during the early implementation phase of ECG acquisition by nonmedical EMS personnel, the effectiveness of the training program was not evaluated. Previous studies have shown that structured ECG training programs can significantly improve ECG acquisition performance and provider confidence [27]. Further studies are needed to assess whether additional training, standardization, or competency reassessment could improve ECG acquisition quality in the prehospital setting.

## 5. Conclusions

More than 70% of prehospital ECGs met predefined technical interpretability criteria; however, these criteria reflect a technical assessment and should not be directly equated with clinical usefulness. The findings should be interpreted as relating to technical acquisition quality only. Any implications for diagnosis, triage, or outcomes remain speculative and require dedicated prospective evaluation. No statistically significant between‐group difference was observed, but the study was underpowered and should not be interpreted as demonstrating equivalence between medical and nonmedical teams. Larger prospective studies are required to better assess clinical impact and comparative performance. This study highlights areas for potential improvement in prehospital ECG acquisition and interpretation. While extending ECG acquisition to nonmedical personnel enhances prehospital care capabilities, improving training, equipment standardization, and the integration of rescue personnel into emergency protocols are essential for better patient outcomes. Larger prospective studies with balanced group sizes are required to adequately assess comparative performance and to formally test equivalence or noninferiority between medical and nonmedical ECG acquisition.

## Funding

This research did not receive any specific funding from any public, commercial, or non‐profit funding organization.

## Conflicts of Interest

The authors declare no conflicts of interest.

## Supporting Information

Additional supporting information can be found online in the Supporting Information section.

## Supporting information


**Supporting Information** Supporting Figure 1: Flow diagram of ECG selection and data processing from the emergency medical dispatch center database. Supporting Figure 2: Educational material used for ECG acquisition training of nonmedical EMS personnel. Supporting Figure 3: STROBE checklist for observational studies. Supporting Figure 4: RECORD checklist for studies using routinely collected health data.

## Data Availability

The data of this study are available upon reasonable request.
