# Supplementary material for: Assessment of the Technical Interpretability of Prehospital ECGs Performed by Medical and Nonmedical Teams in a French Emergency Medical System: A Descriptive Pilot Study
Source: Emerg Med Int. 2026 May 20;2026:7380174. doi: 10.1155/emmi/7380174 (PMC13189496; doi:10.1155/emmi/7380174)
Supplement: Supplementary file 1 — Supporting Information Supporting Figure 1: Flow diagram of ECG selection and data processing from the emergency medical dispatch center database. Supporting Figure 2: Educational material used for ECG acquisition training of nonmedical EMS personnel. Supporting Figure 3: STROBE checklist for observational studies. Supporting Figure 4: RECORD checklist for studies using routinely collected health data. [file EMMI-2026-7380174-s001.docx]

**Supplementary appendix**

**
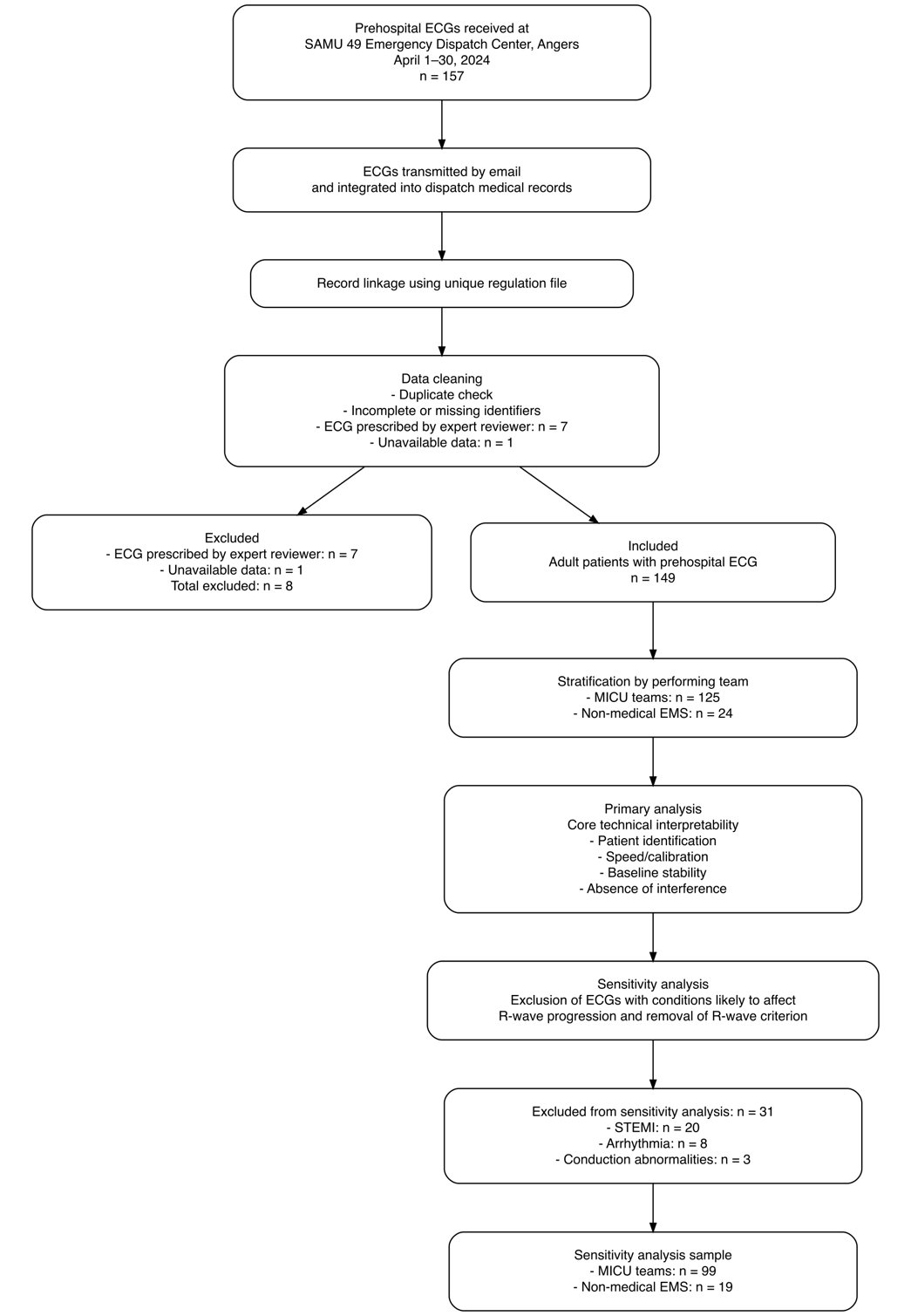
**

**Supplementary Figure 1. Data provenance and selection of prehospital ECGs.** Prehospital ECGs received at the SAMU 49 Emergency Medical Dispatch Center (Angers, France) between April 1 and April 30, 2024 (n = 157) were transmitted by email and integrated into dispatch medical records. ECGs were linked to emergency regulation files using a unique identifier. After data cleaning, 8 ECGs were excluded (7 prescribed by an expert reviewer and 1 with unavailable data), resulting in 149 included adult patients. ECGs were stratified into MICU teams (n = 125) and non-medical EMS (n = 24). The primary analysis assessed core technical interpretability criteria only. A sensitivity analysis excluded ECGs with STEMI, arrhythmia, or conduction abnormalities (n = 31), resulting in 99 MICU and 19 non-medical EMS ECGs.

**
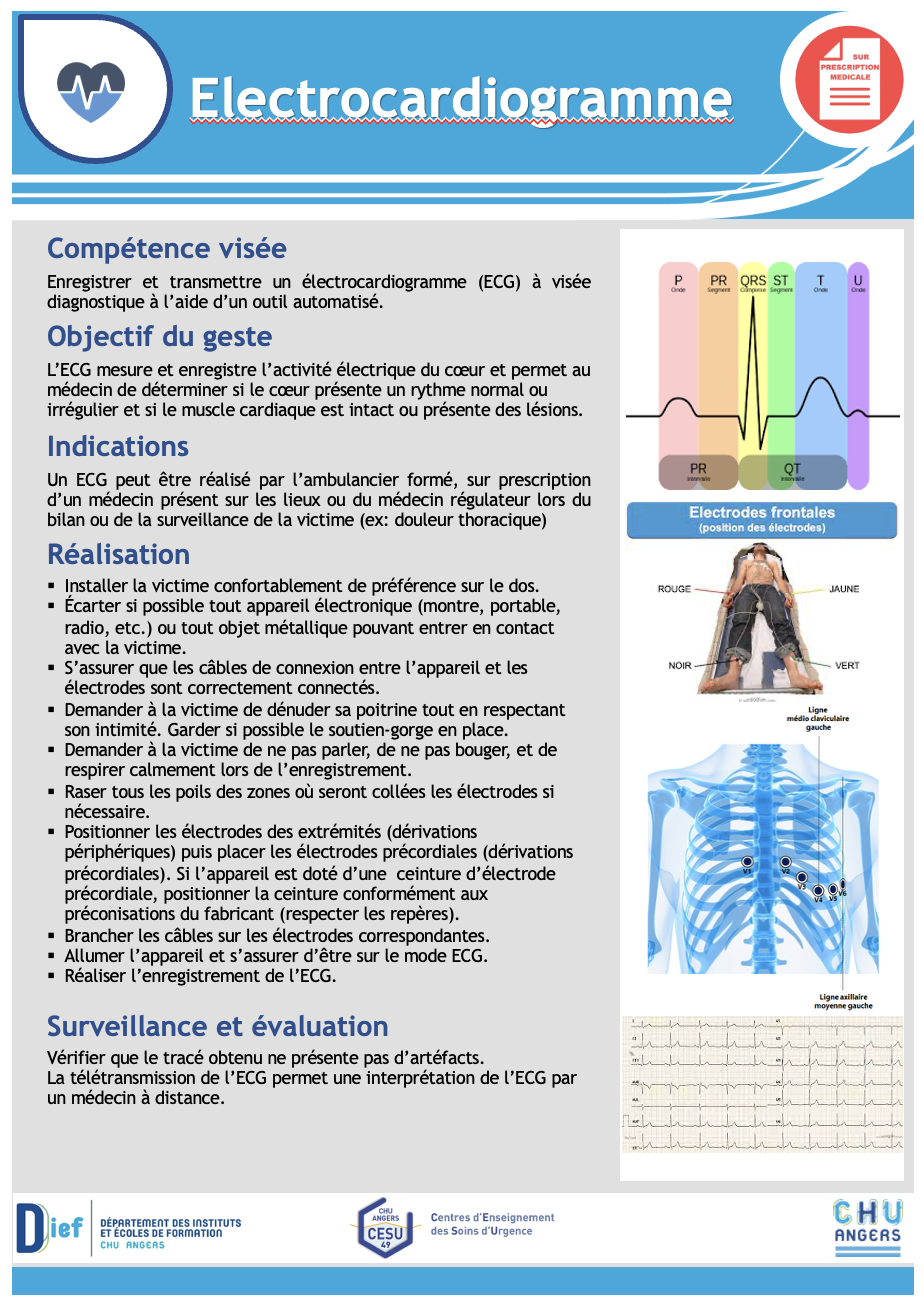
**

**Supplementary Figure 2. Training material for prehospital ECG acquisition.** Educational material used during **the** standardized training program delivered to non-medical EMS personnel (ambulance technicians and firefighters) by the Emergency Care Training Center (CESU49). The figure illustrates key elements of ECG acquisition, including indications, patient preparation, electrode placement, recording procedures, and quality control. This material reflects the standardized framework used to train EMS personnel in prehospital ECG acquisition.

| **Item** | **Recommendation** | **Location in Manuscript** |
| --- | --- | --- |
| Title and abstract | Indicate study design | Title page, Abstract (p.1) |
| Background/rationale | Scientific background | Introduction (p.2) |
| Objectives | Study objectives | Introduction (p.2) |
| Study design | Key elements of design | Methods – Study design (p.3) |
| Setting | Setting and dates | Methods – Setting (p.3) |
| Participants | Eligibility criteria | Methods – Study population (p.3–4) |
| Variables | Define outcomes and variables | Methods – Outcomes (p.4) |
| Data sources/measurement | Data sources and measurement | Methods – Data collection (p.4–5) |
| Bias | Address potential bias | Methods (p.5) + Discussion (p.8) |
| Study size | Explain sample size | Methods (p.5) |
| Quantitative variables | Handling of variables | Methods – Statistical analysis (p.5–6) |
| Statistical methods | Statistical methods | Methods – Statistical analysis (p.5–6) |
| Participants (results) | Numbers at each stage | Results + Figure 2 (p.6) |
| Descriptive data | Participant characteristics | Results (p.6–7) |
| Outcome data | Outcome events | Results (p.7) |
| Main results | Estimates with CI | Results + Figure 3 (p.7–8) |
| Other analyses | Subgroup/sensitivity analyses | Results (p.8) |
| Key results | Summary of findings | Discussion (p.8–9) |
| Limitations | Study limitations | Discussion (p.9) |
| Interpretation | Interpretation of results | Discussion (p.9) |
| Generalisability | External validity | Discussion (p.9–10) |
| Funding | Funding statement | End of manuscript (p.10) |

**Supplementary Figure 3. STROBE Checklist – Observational Study.** Checklist of items recommended by the Strengthening the Reporting of Observational Studies in Epidemiology (STROBE) statement. Page numbers refer to the locations in the manuscript where each item is addressed.

**RECORD Checklist – Routinely Collected Health Data**

| **Item** | **Recommendation** | **Location in Manuscript** |
| --- | --- | --- |
| Data source | Type of data used | Methods – Data source (p.3) |
| Data linkage | Linkage methods | Supplementary Appendix (Suppl. p.1–2) |
| Data cleaning | Cleaning procedures | Supplementary Appendix (Suppl. p.2) |
| Inclusion/exclusion | Selection process | Methods (p.3–4) + Figure 2 (p.6) |
| Variables definition | Definition of variables | Methods (p.4) + Supplementary Appendix (Suppl. p.2–3) |
| Bias related to data | Data-related bias | Discussion (p.9) |
| Missing data | Handling missing data | Methods (p.5) |
| Validation | Data validation | Methods (p.5) |
| Accessibility | Data access statement | End of manuscript (p.10) |
| Transparency | Flow diagram | Figure 2 (p.6) |

**Supplementary Figure 4. RECORD Checklist – Routinely Collected Health Data** Checklist of items recommended by the REporting of studies Conducted using Observational Routinely collected health Data (RECORD) statement. This checklist extends the STROBE guidelines for studies using routinely collected health data. Page numbers refer to the locations in the manuscript or supplementary material where each item is addressed.
